# Childbirth Experiences and Delivery Care During Times of War: Testimonies of Syrian Women and Doctors

**DOI:** 10.3389/fgwh.2021.605634

**Published:** 2021-06-30

**Authors:** Hyam Bashour, Mayada Kharouf, Jocelyn DeJong

**Affiliations:** ^1^Faculty of Medicine, Al Sham Private University, Damascus, Syria; ^2^Faculty of Medicine, Damascus University, Damascus, Syria; ^3^Faculty of Health Sciences, American University of Beirut, Beirut, Lebanon

**Keywords:** maternal health, childbirth, women, war, Syria

## Abstract

**Background:** Until the eruption of violence in 2011, Syria made good progress in improving maternal health indicators including reducing the maternal mortality ratio and increasing the level of skilled birth attendance. The war in Syria has been described as one of the worst humanitarian crises in recent times. Damascus Maternity Teaching Hospital is the largest maternity public hospital in the country that survived the war and continued to provide its services even during periods of pronounced instability. The main aim of this paper is to highlight the experience of childbirth and delivery care as described by women and doctors at times of severe violence affecting Damascus.

**Methods:** This paper is based on secondary analysis of qualitative data collected between 2012 and 2014 for a WHO-funded implementation research project introducing clinical audits for maternal near-misses. This analysis specifically looked at the effects of violence on the childbirth experience and delivery care from the perspective of both women and physicians. A total of 13 in-depth interviews with women who had recently delivered and survived a complication and 13 in-depth interviews with consultant obstetricians were reviewed and analyzed, in addition to three focus group discussions with 31 junior care providers.

**Results:** Three themes emerged concerning the experiences of women and doctors in these times of war. First, both women and doctors experienced difficulty reaching the hospital and accessing and providing the services, respectively; second, quality of care was challenged at that time as perceived by both women and doctors; and third, women and doctors expressed their psychological suffering in times of hardship and uncertainty and how this affected them.

**Conclusions:** Efforts to safeguard the safety of delivery and prevent maternal mortality in Syria continued despite very violent and stressful conditions. Both women and providers developed strategies to navigate the challenges posed by conflict to the provision of delivery care. Lessons learned from the experiences of both women and doctors should be considered in any plans to improve maternal healthcare in a country like Syria that remains committed to achieving the Sustainable Development Goals in 2030 in the aftermath of nearly 10 years of war.

## Introduction

Syria has achieved good progress in achieving the Millennium Development Goals (MDGs), including those related to maternal health, as was reflected in the third national MDG report of 2010 (1). The war in Syria that erupted in 2011 has resulted in devastating destruction across the entire range of basic services and infrastructure. In several governorates, hospitals, clinics, health centers, and schools have been destroyed, with many more that are only partly functional. The WHO HeRAMS (Health Resources and Services Availability Monitoring System) data indicated that out of the 113 assessed public hospitals in the country, 50% were reported fully functioning by the end of 2019, 25% were reported partially functioning (i.e., shortage of staff, equipment, medicines, or damage of the building in some cases), while 25% (28 hospitals) were reported non-functioning (2). The war has also resulted in the huge displacement of the Syrian population seeking protection, whether as refugees or internally displaced. The first national Sustainable Development Goals (SDGs) report indicated that by 2015, 63.9% of the population was living below the poverty line (3). In spite of the dynamic situation in some parts of the country, however, the rehabilitation and restoration of infrastructure and services in most of the country have started. Needs are being assessed, and measures are being taken by the government and UN partners (4).

Prior to 2011, 96% of Syrian women delivered with the help of a skilled birth attendant, and 88% had at least one antenatal care visit (5). The maternal mortality ratio (MMR) just prior to the war was estimated nationally at 52/100,000 live births in 2009 (5). During the years of war, the MMR has increased with an estimate of 68/100,000 live births in 2015 being the only available figure from the WHO/UNICEF (6). It is worthwhile to note that the first national SDG report has set the MMR target in the country at 45/100,000 by 2030 (3). According to a large population-based study carried out in 2005, the main cause of maternal death in Syria was hemorrhage (accounting for 65% of maternal deaths) (7). About 91% of maternal deaths was deemed to be preventable in that study through improved clinical care.

Before the war that began in 2011, Syrian women suffered from problems beyond access to services, ranging from failure to take preferences of women at childbirth into account (8), and excessive levels of medical interventions, such as cesarean section (9). Syrian women reported that they preferred to be attended by a female doctor (8). Many additional maternal health problems emerged or were exacerbated during the war period. For example, a recent study on the prevalence of cesarean section at Damascus Maternity Teaching Hospital during the Syrian war found that the C-section rate had increased from 29% in 2010 (11,939 deliveries) to 46% in 2016 (12,481 deliveries) and further to 51% in the first half of 2017 reported out of 5,278 deliveries (10).

According to available data, coverage rates of most key evidence-based interventions in reproductive, maternal, newborn, and child health have declined in Syria due to the war (11). Although the reasons for the decline have not been extensively studied, likely causes are the lack of access to health facilities, which have been destroyed/disrupted, an exodus of healthcare professionals on the supply side and on the “demand” side, the increased poverty rate—affecting women and their access to care for themselves and their families (11). There have also been increasing risks of outbreaks of previously controlled epidemics (11). Akik et al. reported that the security situation in the country has influenced health-seeking behaviors of populations in Syria (12). Efforts to rebuild the health system in Syria are likely to focus on addressing supply-side factors that are related to governance, infrastructure, rebuilding the destroyed health facilities, and retaining the health workers with the overall aim of achieving universal health coverage (13). However, little is known about the expectations and experiences of health workers who are at the core of any health service, particularly in the case of maternal health.

The main aim of this paper is to highlight the experience of childbirth and delivery care as described by women and doctors at times of severe violence affecting Damascus city, the capital of Syria, during the period 2012–2014.

## Methods

The overview of qualitative methods below has been checked against the Consolidated Criteria for Reporting Qualitative Studies (COREQ) guidelines (14).

### Study Design

We conducted a secondary analysis of qualitative data collected in a WHO-funded implementation research project aimed to assess the acceptability, feasibility, and effectiveness of clinical audits for maternal near-miss cases. The secondary analysis is the use of existing data to find answers to research questions that differ from the questions asked in the original research (15). This secondary analysis specifically looked at the effects of violence and security issues on the childbirth experience and delivery care. The findings of the original quantitative research have been published (16), but the analysis of the qualitative data of the original research has not yet been published.

To obtain views of providers on the health system constraints and opportunities to providing safe and quality care in delivery, in-depth interviews were carried out with obstetrician–gynecologists purposively selected according to characteristics likely to affect their perceptions of quality of care, namely seniority, position, and length of time working in a hospital. Post-intervention (a quality improvement program implementing clinical audits for the management of near-miss cases), key informant interviews with the hospital director and senior doctors were also carried out to elicit their views on the intervention and the challenges seen during the implementation of the project. In addition, focus group discussions (FGDs) with junior doctors were conducted using a discussion guide focusing on their perceptions of the intervention strategies and potential barriers, challenges, and mechanisms to ensure sustainable implementation. On the other hand, women who survived the complication (near miss^1^) were interviewed to elicit their views on the experience and satisfaction with care providers.

### Study Setting

This study took place at Damascus Maternity Teaching Hospital, which is the largest maternity teaching hospital in the country. In 2011, and prior to the eruption of violence in the country, the 250-bed hospital established in 1983 had ~20,000 admissions yearly, 60% of which were accepted in labor and pregnancy units. The hospital continued to provide its services throughout the war years with an average of 11,000–13,000 deliveries per year. Damascus city was particularly badly affected between 2013 and 2015, and this affected access to the hospital.

### Sample

Near miss women were recruited for the study along with a purposive sample of care providers. A total of 13 out of 18 near miss women who nearly died but survived a complication were interviewed. All consultant obstetricians (a total of 13; 11 male and 2 female) were interviewed. Another 31 junior doctors (17 male and 14 female) were invited to take place in the FGD. As nurses and midwives at the hospital do not directly provide care for the near-miss cases, they were not included in this study. However, they took part in the audit meetings in our original research.

### Data Collection

Face-to-face, in-depth interviews were conducted for the primary research on the experience of women with near miss. Interviews were conducted by a female Syrian researcher (a member of the research team) with a Master's degree in Anthropology. The interviews took place at the hospital at the convenience of the women, assuring privacy. The average interview duration was ~30 min.

The same researcher interviewed the senior consultants using an in-depth interview guide at times convenient for the consultants. Interviews were recorded if audio recording was accepted by the participant.

Another experienced qualitative female researcher with a Master's degree in Sociology carried out the three FGD with the junior residents. A discussion guide was used. The FGDs took place at the hospital in a large designated room and took on average 2 h. A trained assistant attended the FGDs and took notes. The fieldwork was conducted during 2013–2014.

### Data Analysis

Two of the authors read all available transcripts and notes from the original research. Audio recordings in Arabic were listened to sequentially, and segments were included or excluded according to whether they contained any comments or views related to the effect of war on maternal care. Each transcript was read by two of the authors individually who made notes when interesting or relevant information is found. Data were coded through repeated review of the transcripts linked to the research questions. Thematic coding captured important issues, connections, and clarifications that helped in explaining similar statements. Themes were then discussed and agreed upon between the two researchers.

### Ethics

The original research protocol was reviewed by the WHO specialist panel and approved by the WHO Research Ethics Review Committee (ERC). The study was also approved by the ERC at Damascus University and the Institutional Review Board (IRB) of the American University of Beirut as the coordinating institution. Written consent from all participants was obtained. For the secondary analysis reported here, no submission was made to an ethical committee as the researchers themselves did the analysis with the data available from their earlier study.

## Results

Three dominant themes emerged throughout data collection with women, senior obstetricians, and junior doctors who characterized their experiences of childbirth and caregiving at a time when violent conflict was pervasive in Damascus. These were mainly related to: first, difficulty of women and doctors in reaching the hospital and accessing and providing the services, respectively; second, a perception by both women and doctors whose quality of care was compromised; and third, both women and doctors expressed psychological suffering in times of hardship and uncertainty at the time of data collection and how this affected them.

### Theme 1: Access to the Hospital by Women and Doctors

The difficulty in reaching the hospital whether to receive care by women or to provide care was a major issue described by the research participants, whether women or providers. Women self-referred themselves and went directly to hospitals, noting that according to the inclusion criterion of the original study they were near miss cases with high-risk pregnancies and complications. Women described their critical cases as being the driver to be self-referred to a hospital. Due to the security situation in the capital, women and families sought care at hospitals close to their homes. However, refusal by hospitals sometimes occurred due to lack of availability of specialized doctors or needed facilities for critical care. Women were then transferred again by ambulance cars owned by the hospitals or by the central ambulance service to Damascus Maternity Teaching Hospital.

One woman, for example, a resident of a location slightly far from Al Baramkeh, where the teaching hospital is located, described going with her family to a nearby hospital by taxi “*…They did not receive me at the nearby hospital. They transferred me to Dar Tawleed Hospital (The known name of the Teaching Hospital). They said it is better there for my case. We were afraid but because of the availability of the ambulance, I was transferred by the ambulance… It is better by the ambulance. Things are quicker and easier.”*

Another woman explained her experience having selected a close hospital to her home where the family felt it was safer since she was bleeding a lot and needed blood transfusion. Her husband went to fetch the blood from the central blood bank (around 7 km) but due to his delay on the road, the hospital transferred the woman by ambulance to the Teaching Maternity Hospital, being the main referral hospital. She said: “*You know they transferred me—out out- without the knowledge of my husband. It will take time for him on the road due to the checkpoints and thus they decided to refer me to this hospital by the fast ambulance car. They called him by phone and told him to go to Dar Al Tawleed. He was surprised and shouted why and they explained that I was bleeding a lot.”*

On the other hand, one woman explained that this is her first time coming to this hospital. It is not her usual place of delivery, but due to the stressful living conditions and the difficult socioeconomic situation, they had to select Dar Al-Tawleed since it is a free public hospital. They were not able to afford the usual moderately priced private maternity hospital. The woman said: “*My private doctor told me that I need to deliver in a hospital because of the bad anemia I have. Due to our situation, she advised me (the doctor) that this teaching hospital is a good choice as it is free since my condition is bad.”*

The issue of their own access to the hospital was also raised by the senior doctors (who tend to live independently from the hospital) but less by the junior doctors who usually reside in the hospital. Senior doctors explained their frustration of having to come every day to the hospital when the security situation was bad. Apart from the risk everyone was under due to the mortars being randomly thrown to the city by the armed militias, they were also annoyed by the long wait at the checkpoints set up by the government. The number of checkpoints they had to cross, and the associated waiting time differed according to the area of residence of the doctors. Some who had to commute long distances to the city had to pass as many as three checkpoints on the drive to or from the hospital.

The difficult and challenging situation was described by the hospital director who said: “*You know how difficult the situation is. We had to face problems. We were flexible with the doctors of course. And we had to find alternatives. We dealt with the situation case by case… it is a lot of responsibility for doctors themselves and for the patients.”* Another doctor noted the issue of checkpoints and its impact. “*Due to the checkpoints and the length of time to reach the hospital, we received many complicated cases that deteriorated on the road. Though the checkpoints are necessary for our safety but delay in transfer of critical cases is happening even if women were transferred by an ambulance”* (Senior Doctor No. 3).

### Theme 2: Quality of Care Challenged

Under the conditions under which the Maternity Teaching Hospital was functioning during the time of data collection, both women and providers expressed that there were challenges to receiving and providing, respectively, quality care. The limited availability of supplies was the main issue cited. When asked about their satisfaction with the care they received, women (though in critical stage) were largely happy. They expressed general satisfaction with the care although some expressed their frustration with the treatment they received from the nurses or with the policy of the hospital not allowing a companion with them.

One woman said: “*Haram (an expression of sympathy in Arabic) the care they provide is good. I am lucky now. I am good. But I wish they allowed my sister in. They do not allow companions. Companions can give you a hand and assist you. It is not allowed here*.”

Another woman said: “*Luckily in this hospital blood is available. In the private hospital I went to, blood was not available and my husband went to fetch it from the central blood bank. This is a large hospital. But, still I asked the nurse to change my dirty gowns but she did not bring me any. Indeed I had to ask one doctor for a clean gown and he provided me with one*.”

Doctors, however, were in better position to describe the limited capacity of the hospital at the time, where resources were very limited due to the situation in the country. Medicines were very scarce, and equipment in need of repair often stopped working, as there were external sanctions on the country. The hospital director said: “*I would say that the crisis has brought good impact on the hospital!! Here we are providing quality care to a large number of patients under very limited resources. This needs to be stated loudly*.”

One senior doctor said: “*The war has impacted our work a lot. The cesarean sections increased a lot and thus we are seeing many complications now. Further, there are shortage in chemotherapy for cancer patients. The drugs used to be provided to patients but now and due to its high cost and the sanctions, they are not available*.” When asked why the cesarean sections increased, he listed several reasons including the high demand of women to deliver during the day hours as to avoid nighttime delivery due to the security situation. Doctors also preferred to reduce the time of delivery as the situation can be risky for doctors as few doctors were kidnapped at night.

In one FGD, the junior doctors noted that things had become very different during the war compared to before the war and that many deaths occurred due to the lack of blood and other emergency equipment.

### Theme 3: Psychological Suffering

It is well-known that war brings mental distress and suffering to the general population. However, our analysis identified psychological distress as it has been largely expressed by doctors due to the hardships they were under and that varied between senior doctors and junior doctors who participated in the FGD.

One senior doctor said: “*The pressure of work has increased a lot as our hospital is now one of two public hospitals in Damascus and surroundings. We are working under the pressure of limited supplies, lack of blood and medicine, sanctions, and more. All these added to our responsibility to follow up our patients when access to hospitals is difficult. We are under a lot of stress; you see.”*

Junior doctors described their frustration as well. They described their feelings where the pressure of work is huge due to the high patient load and the stressful conditions. Furthermore, they were also afraid of the security situation. Mortar attacks affected the hospital area and the area next to the university, raising constant fear of attacks. One junior doctor said: “*We are tired. We are under a lot of pressure.”*

Another young resident noted that there is also the stress of conflict itself. Due to the conflict situation, there were variations in views regarding politics and tactics. He said (sarcastically): “*We love each other a lot”* referring to the conflict happening between individuals expressing different attitudes to things around them.

On the other hand, junior doctors also discussed the effect of psychological distress on the health of women. They claimed that they are seeing a lot of cases of premature labor due to the stress experienced by the pregnant women.

## DISCUSSION

Overall, this paper analyzed perspectives of women seeking care at the Damascus Maternity Teaching Hospital and perspectives of senior and junior doctors providing care at the hospital at a time when the security situation was badly affected in Damascus after the eruption of violence in 2011.

It was not surprising to see that the main issue experienced by women and doctors was the inaccessibility to the hospital. Many other authors have reported on the point of accessibility of care in conflict-affected countries (18–20). Damascus at the time of data collection (2012–2014) was not safe due to random mortars hitting the city and many governmental checkpoints in place. These direct security issues imposed high levels of pressure on women who reported self-referring themselves to nearby small hospitals, where they could not receive the care they needed due to their complicated condition. They were then transferred to a large teaching referral hospital by ambulance. This caused delays, which compromised their health and their chances of survival. Fortunately, all of our interviewees were near misses; in other words, they survived the complication they experienced. However, this suggests that there is a dearth of information on those who did not survive.

Doctors clearly admitted the negative impact of delay on the survival of the women in the hospital as they referred to more deaths occurring there. Moreover, they themselves suffered the nuisances of delays reaching their duty at the hospital, which included frustration with the drive and the risk they faced at any moment to their life added to the time showing their identification cards at the checkpoints. The above not only affected the survival of women but also had an impact on the mental health and well-being of the doctor. Apparently, junior doctors who are usually residents in the hospital felt a lot of pressure due to their work overload at the hospital. The high pressure and the state of mental health of doctors were dominant issues in the three FGDs we held. No differences were noted between male and female doctors.

The above issue of insecurity and its implications on women or even health workers was described and discussed in a recent article on delivering health interventions for women in conflict settings. Syria was one of 10 case studies that were described in that article (21).

On another note, from the leadership point of view of the hospital, there was a sense of satisfaction as the hospital continued to provide its services to the women, and even the number of admissions increased sometimes, as it was one of only two public hospitals providing free maternity services in Damascus. The other two public hospitals in and around Damascus were partly destroyed and not functioning. According to WHO HeRAMS, Comprehensive Emergency Obstetric Care (CEmOC) was available in 74% of the functioning public hospitals in the country in the third quarter of 2013 (22).

The hospital leadership admitted the hard conditions in which the hospital was functioning. There was a lot of shortage in supplies, including medicines, equipment, and others lifesaving blood products. The shortage in medicines was due to many factors including the destruction of the extensive pharmaceutical industry in the country during the war. Shortage of blood was more severe in small private hospitals where women sought care prior to being admitted to our study hospital. Syria prewar was producing 95% of its needed medicines (23). Other reasons included the shortage of funds during the war and the complexities of importing supplies given the economic situation, and that the country was under unilateral sanctions and still is (24, 25). Sanctions were pinpointed by the doctors as the main reason hampering the repair of medical equipment in the hospital.

Damascus Teaching Hospital was the site of several studies carried out by the co-authors who are members of a long-standing network of regional researchers called Choices and Challenges of Changing Childbirth (26) and the Reproductive Health Working Group (27). Reports of the poor maternal quality of care in Middle Eastern Countries including our hospital have been published by network members (28). This was the reason for the original research on the effectiveness of clinical audits for which the qualitative data were conducted.

A recurrent theme that came out in the findings of our study was the concerns of women over the quality of care, particularly in relation to their need for companionship during labor and delivery; separate intervention studies have been carried by network members with the aim to address this limitation of maternal health policy in Syria (29). It is important to note that allowing trained companions is an easy-to-implement and low-cost intervention that might be even more critical in times of war to provide support and that could potentially reduce pressure on physicians. Findings from the WHO reproductive, maternal, newborn, child, and adolescent health policy survey carried out in 2018 (30) indicated that 12 out of 16 countries in the WHO Eastern Mediterranean Region do not have policies to recommend the presence of a companion of choice at labor and birth (31). Moreover, the issue of poor intrapersonal communication and bad treatment by staff was previously reported, yet again emerged in our current work (32). Much of those prewar conditions carry implications for the satisfaction with the care women receive during the war period. But we were not able to study this in full depth in our work in terms of how the war might have possibly worsened those issues. From the point of view of providers, it was mainly the shortage of supplies and the delays in referral to hospitals that affected the quality of care at the time of war.

It is worthwhile noting the observation of young doctors we interviewed about the considerable psychological stress that women suffered and its relationship with preterm delivery. The results of a recent verbal autopsy study by the Syrian Ministry of Health and UNICEF, which was used to identify the causes of death in under-five mortality (33) indicated that the first cause of neonatal deaths was prematurity accounting for 43% of deaths among neonates <4 weeks. How much of this can be correlated to stress or other factors influenced by the war is still to be understood.

The strength of this work is that it took place in Damascus Maternity Teaching Hospital, which has been the site of many studies led by the first author including two WHO implementation projects that were implemented place during the war years 2012–2017. The familiarity the authors have with the setting adds depth to the interpretation of the findings. The qualitative data have provided us with a good understanding of the experiences of women and doctors at a time where statistics and quantitative data were limited, and most of the reports came from humanitarian agencies. However, the main limitation is that this work is based on the secondary data analysis of qualitative data collected from an earlier three countries study on the management of maternal near-miss cases, as described above. Yet again, in the Syrian context, the interview guide at the time of primary data collection included a question on the impact of the crisis on the possibility to implement or sustain the clinical audit in the context of Damascus Maternity Teaching Hospital or other hospitals in the country (unlike the case in the studies in other participating countries in the research, namely Lebanon and Egypt). Nevertheless, assessing the impact of conflict on maternal health was not the primary objective of the data reported here. The secondary data analysis has been criticized by researchers (34, 35) for this reason, and we concur that more in-depth and primary research would have been of interest to scholars had it been possible. However, it should be noted that conducting research at that time, and obtaining research funding to do so, was extremely challenging.

Conflict-causing factors are not only limited to resources (or lack thereof), economic, or otherwise. The results of the current study have important implications on the provision of care in times of conflict. Understanding the barriers facing pregnant women in complex emergencies is the first step to ending suffering and maternal deaths—and creating a more resilient society. The same applies to providers of care where resilience was pushing them to provide care despite their tremendous psychological suffering in stressful and risky working conditions.

## Data Availability Statement

The data analyzed in this study is subject to the restrictions due to the confidential nature of the qualitative data and interviews in Arabic. Requests to access these datasets should be directed to Hyam Bashour, hyam1929@gmail.com.

## Ethics Statement

The studies involving human participants were reviewed and approved by WHO Research Ethics Review Committee, The ERC at Damascus University, and The Institutional Review Board (IRB) of the American University of Beirut. The patients/participants provided their written informed consent to participate in this study.

## Author Contributions

HB and JD initially designed the original research protocol. HB and MK supervised the original fieldwork and done the secondary analysis of the qualitative data. HB drafted the manuscript. MK and JD helped in drafting the manuscript. All authors contributed to the conception of this manuscript, revised the final manuscript, and approved it.

## Conflict of Interest

The authors declare that the research was conducted in the absence of any commercial or financial relationships that could be construed as a potential conflict of interest.

## Footnotes

^1^Maternal Near Miss (MNM) is defined by the WHO as a woman who nearly died but survived a complication that occurred during pregnancy, childbirth, or within 42 days of termination of pregnancy (17).

## References

[1] Syrian State Planning Commission. Third National MDGs Progress Report 2010, Damascus. (2010). Available online at: http://www.undp.org/content/dam/rbas/report/MDGR-2010-En.pdf (accessed May 11, 2021).

[2] WHO. HeRAMS Public Hospitals Report –Syria. (2019). Available online at: http://www.emro.who.int/syr/information-resources/herams-reports.html (accessed May 11, 2021).

[3] Syrian State Planning Commission. First National SDGs Progress Report 2019, Damascus. (2019). Available online at: https://www.arabdevelopmentportal.com/publication/first-national-report-sustainable-development-goals-sdgs (accessed May 11, 2021).

[4] UN. Humanitarian Response Plan for the Syrian Arab Republic. (2019). Available online at: https://reliefweb.int/sites/reliefweb.int/files/resources/2019_hrp_syria.pdf (accessed May 11, 2021).

[5] Syrian Central Bureau of Statistics. League of Arab States. Family Health Survey in Syrian Arab Republic – 2009. Damascus (2011).

[6] WHO/EMRO. Syria Country Profile. (2017). Available online at: https://rho.emro.who.int/sites/default/files/Profiles-briefs-files/Syria-02.pdf (accessed May 11, 2021).

[7] Bashour H Abdulsalam A Jabr A Cheikha S Tabbaa M Lahham M. Maternal mortality in syria: causes, contributing factors and preventability. Trop Med Int Health. (2009) 14:1122–7. 10.1111/j.1365-3156.2009.02343.x 19624475

[8] Bashour H Abdulsalam A. Syrian women's preferences for birth attendant and birth place. Birth. (2005) 32:20–6. 10.1111/j.0730-7659.2005.00333.x 15725201 PMC1457105

[9] Khawaja M Kabakian-Khasholian T Jurdi R. Prevalence and determinants of c-section in Egypt: evidence from DHS. Health Policy. (2004) 69:273–81. 10.1016/j.healthpol.2004.05.006 15276307

[10] Al-Hammami H Taleb MJ Alsharif MN Taleb MM. Prevalence of Cesarean Section at ALTAWLID Hospital during the Syrian Crisis. J Med Pharma Allied Sci. (2017) 6:947–53. 10.22270/jmpas.v6i12.746

[11] DeJong J Ghattas H Bashour H Mourtada R Akik C Reese-Masterson A. Reproductive, maternal, neonatal and child health in conflict: a case study on Syria using Countdown indicators. BMJ Glob Health. (2017) 2:e000302. 10.1136/bmjgh-2017-000302 29225945 PMC5717942

[12] Akik C Semaan A Shaker-Berbari L Jamaluddine Z Saad GE Lopes K. Responding to health needs of women, children and adolescents within Syria during conflict: intervention coverage, challenges and adaptations. Conflict Health. (2020) 14:37 10.1186/s13031-020-00263-3 PMC7278078 32523615

[13] Abbara A Blanchet K Sahloul Z Fouad F Coutts A Wasim M. The effect of the conflict on Syria's health system and human resources for health. World Health Populat. (2015) 16:87–95. 10.12927/whp.2015.24318

[14] Tong A Sainsbury P Craig J. Consolidated criteria for reporting qualitative research (COREQ): a 32-item checklist for interviews and focus groups. Int J Q Health Care. (2007) 19:349–57. 10.1093/intqhc/mzm042 17872937

[15] Hinds PS Vogel RJ Clarke-Steffen L. The possibilities and pitfalls of doing a secondary analysis of a qualitative dataset. Qualit Health Res. (1997) 7:408–24. 10.1177/104973239700700306

[16] Bashour H Saad-Haddad G DeJong J Ramadan MC Hassan S Breebaart M. A cross sectional study of maternal ‘near-miss' cases in major public hospitals in Egypt, Lebanon, Palestine and Syria. BMC Pregnancy Childbirth. (2015) 15:296. 10.1186/s12884-015-0733-7 26566955 PMC4644334

[17] Say L Souza JP Pattinson RC. Maternal near miss - towards a standard tool for monitoring quality of maternal health care. Best Pract Res Clin Obstet Gynaecol. (2009) 23:287–96. 10.1016/j.bpobgyn.2009.01.007 19303368

[18] Christina CM. Women and access to healthcare. Soc Sci Med. (1992) 35:619–26. 10.1016/0277-9536(92)90356-U 1381522

[19] Usta J Farver JAM Zein L. Women, war and violence: Surviving the experience. J Women's Health. (2008) 17:793–804. 10.1089/jwh.2007.0602 18537482

[20] Degni F Amara I Klemetti R. Women's experiences in accessing maternal and child health services during the period of the armed conflict in the North of Mali. Open Public Health J. (2015) 8:17–22. 10.2174/1874944501508010017

[21] Singh NS Ataullahjan A Ndiaye K Das JK Wise PH Altare C. Delivering health interventions to women, children, and adolescents in conflict settings: what have we learned from ten country case studies? Lancet. (2021) 397:533–42. 10.1016/S0140-6736(21)00132-X 33503459

[22] WHO. HeRAMS Public Hospitals Report –Syria. (2013). Available online at: http://www.emro.who.int/syr/information-resources/herams-reports.html (accessed May 11, 2021).

[23] Central Bureau of Statistics. Statistical Abstract 2019, Damascus (2019). Available online at: http://cbssyr.sy/ (accessed May 11, 2021).

[24] Al Faisal W Hussain H Sen K. Perilous path to peace in the Middle East: the dilemma of sanctions. In: Barrett DH, Ortmann LW, Dawson A, Saenz C, Reis A, eds. Public Health Ethics: Cases Spanning the Globe. Melbourne, VIC: Springer Open (2018). p. 274–79.

[25] Hussain H Sen K. EU guidance impedes humanitarian action to prevent COVID-19 in Syria. Lancet Glob Health. (2020) 8:e1112–3. 10.1016/S2214-109X(20)30289-8 32622402 PMC7332264

[26] Choices and Challenges in Changing Childbirth Research Network. Routines in facility-based maternity care: evidence from the Arab World. BJOG: Int J Obstetr Gynecol. (2005) 112:1270–6. 10.1111/j.1471-0528.2005.00710.x PMC1457116 16101607

[27] Giacaman R Al-Ryami A Bashour H DeJong J Gaballah N Ghressi A. Importance of research networks: the reproductive health working group, Arab world and Turkey. Lancet. (2014) 383:483–5. 10.1016/S0140-6736(13)62704-X 24452048

[28] Bashour H Abdulsalam A Al Faisal W Cheikha S. The patterns and determinants of maternity care in Damascus, Syria. East Mediterr Health J. (2008) 14:595–604. 18720624

[29] Kabakian-Khasholian T Bashour H El-Nemer A Kharouf M Elsheikh O. Implementation of a labour companionship model in three public hospitals in Arab middle-income countries. Acta Paediatr. (2018) 107(Suppl. 471):35–43. 10.1111/apa.14540 30570794

[30] World Health Organization. Reproductive, Maternal, Newborn, Child and Adolescent Health Policy Survey. Geneva: WHO (2018).

[31] World Health Organization. Companion of Choice During Labour and Childbirth for Improved Quality of Care. Evidence-to-Action Brief . Geneva (2020).

[32] Bashour HN Kanaan M Kharouf MH Abdulsalam AA Tabbaa MA Cheikha SA. The effect of training doctors in communication skills on women's satisfaction with doctor–woman relationship during labour and delivery: a stepped wedge cluster randomised trial in Damascus. BMJ Open. (2013) 3:e002674. 10.1136/bmjopen-2013-002674 23945729 PMC3752047

[33] Mauthner N Parry O Backett-Milburn K. The data are out there, or are they? Implications for archiving and revisited qualitative data. Sociology. (1998) 32:733–45. 10.1177/0038038598032004006

[34] Long-Sutehall T Sque M Addington-Hall J. Secondary analysis of qualitative data: a valuable method for exploring sensitive issues with an elusive population‘? J Res Nursing. (2010) 16:335–44. 10.1177/1744987110381553

[35] Syrian Ministry of Health. Causes of Mortality among Children Under Five Years. Damascus (2019).

